# Silicon Derived from Glass Bottles as Anode Materials for Lithium Ion Full Cell Batteries

**DOI:** 10.1038/s41598-017-01086-8

**Published:** 2017-04-19

**Authors:** Changling Li, Chueh Liu, Wei Wang, Zafer Mutlu, Jeffrey Bell, Kazi Ahmed, Rachel Ye, Mihrimah Ozkan, Cengiz S. Ozkan

**Affiliations:** 1 Materials Science and Engineering Program, Department of Mechanical Engineering, University of California, Riverside, CA 92521 USA; 2 Department of Electrical and Computer Engineering, Department of Chemistry, University of California, Riverside, CA 92521 USA

## Abstract

Every year many tons of waste glass end up in landfills without proper recycling, which aggravates the burden of waste disposal in landfill. The conversion from un-recycled glass to favorable materials is of great significance for sustainable strategies. Recently, silicon has been an exceptional anode material towards large-scale energy storage applications, due to its extraordinary lithiation capacity of 3579 mAh g^−1^ at ambient temperature. Compared with other quartz sources obtained from pre-leaching processes which apply toxic acids and high energy-consuming annealing, an interconnected silicon network is directly derived from glass bottles via magnesiothermic reduction. Carbon-coated glass derived-silicon (gSi@C) electrodes demonstrate excellent electrochemical performance with a capacity of ~1420 mAh g^−1^ at C/2 after 400 cycles. Full cells consisting of gSi@C anodes and LiCoO_2_ cathodes are assembled and achieve good initial cycling stability with high energy density.

## Introduction

Green, reliable and energy-efficient lithium ion storage platforms with fast rate capability, high energy density and high power density are essential for the new generation of electric vehicles (EVs) and plug-in hybrid electric vehicles (PHEVs)^1, 2^. Conventionally graphite-based anodes used in commercial lithium ion batteries (LIBs) have a limited theoretical capacity of 372 mAh g^−1^ due to the inadequate Li-ion intercalation in LiC_6_. Silicon is extensively considered the most encouraging material for the next generation anodes owing to the low discharge potential (~0.1 V vs. Li/Li^+^) and the high theoretical capacity of 3572 mAh g^−1^ corresponding to the formation of Li_15_Si_4_ phase at room temperature^3, 4^. If commercially used LiCoO_2_ (~145 mAh g^−1^) is assembled as the common cathodes, the full cells based on Si anodes lead to a 34% increase in the total capacity over that of graphite-anode based full cells^5, 6^. However, Si is able to alloy with a large amount of Li-ions during lithiation, resulting in a large volume expansion upwards of 300%^7^. The lithium-induced mechanical stresses during alloying with subsequent contraction during dealloying can cause Si to fracture, which promote the pulverization of active materials and the deterioration of the conductive network. The repeated expansion and shrinkage during lithiation and delithiation destroy the integrity of solid electrolyte interphase (SEI), while increasing the decomposition of electrolyte to reform SEI on the newly exposed Si surface^8, 9^.

To remedy the above problems, various strategies have been utilized on alleviating the structural volume change and optimizing the electrochemical performance of Si anodes. Downscaling the dimensions of silicon structures has been verified to be an effective path to mitigate the capacity fading stemming from the cracking of Si during lithiation and delithiation^10, 11^. Well-designed nanostructures, such as Si nanoparticles, double walled Si nanotubes, and three-dimensional (3D) porous nano-Si have all been proven to be advantageous in efficiently modifying the volume expansion of Si via the void spaces generated by their porous or hollow structures^12–14^. Moreover, the incorporation of electronically conductive coatings across Si is an effective strategy to improve the cycling stability of Si anodes. Carbon coatings via thermal decomposition of carbon precursors act as soft buffer layers to accommodate the volume expansion of Si^15, 16^. *In-situ* polymerized conductive polymer coverings with tunable conductivity, diverse monomer chemistry and surface compatibility with electrolyte function as conductive shell-matrixes to enhance the rate capability of the electrodes^17–21^.

**Figure 1:**
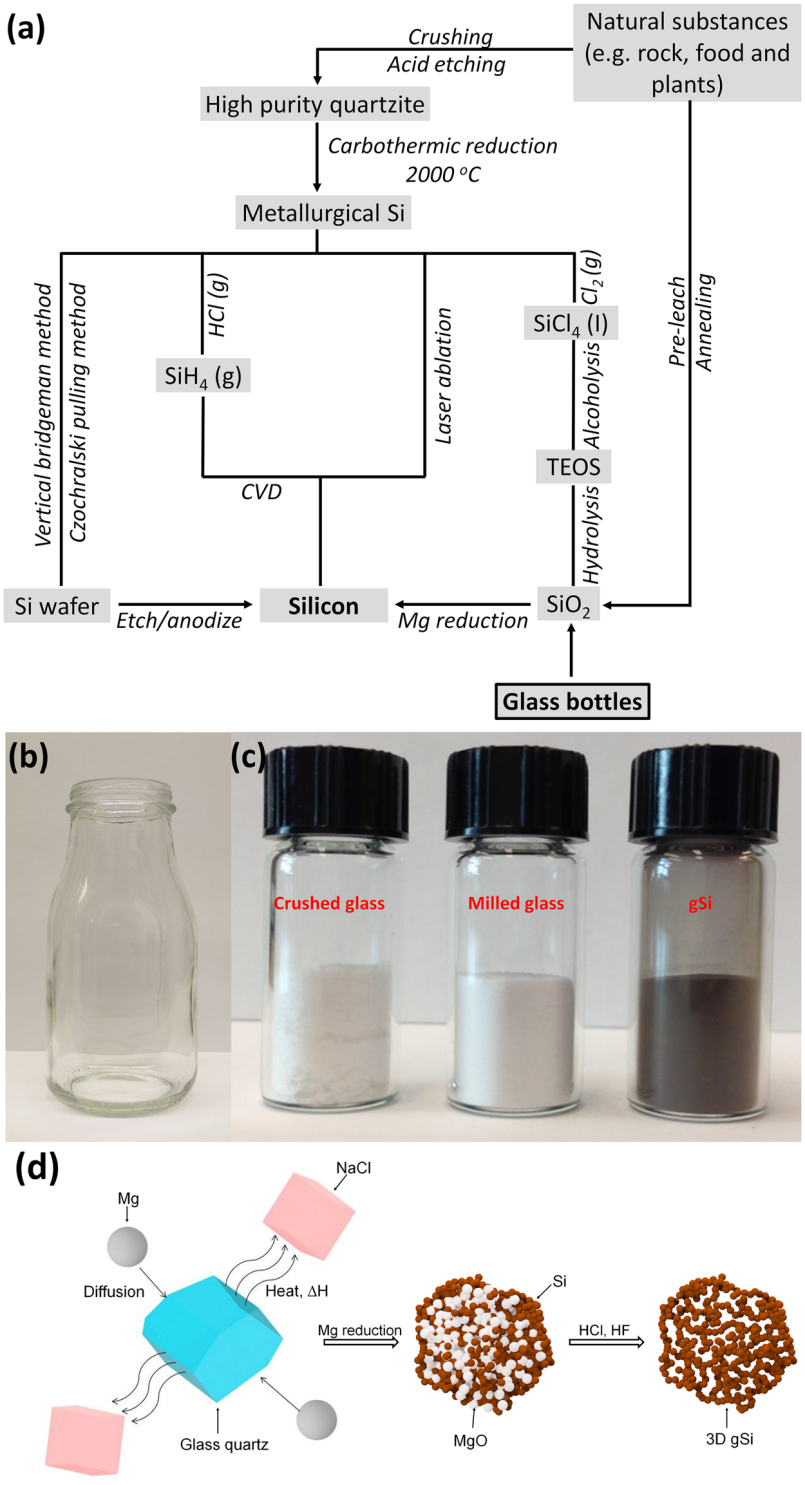
(**a**) Flow chart showing existing synthesis routes for nano-Si, including our synthesis method from glass bottles. (**b**) A collected beverage glass bottle. (**c**) (From left to right) vials of crushed glass, milled glass and gSi powder. (**d**) Schematic of the Mg reduction process using NaCl as heat scavenger.

While a large number of routes for designing nanostructured Si with excellent electrochemical performance as anode materials have been established, many methods for synthesizing Si nanostructures are mainly limited to the costly raw materials, complex procedure and the low yield of active martial as shown in Fig. 1a ^22, 23^. The pyrolysis of silane/halo-silane/polysilane precursors via chemical vapor deposition (CVD) can produce various nanostructured silicon, such as nanospheres, nanowires and nanotubes^24–26^. The electrodes based on these structures show stable cycling and high-rate capability. However, the pyrolysis process consumes a large amount of energy and requires expensive and highly toxic precursors, which make it non-economical and impractical for mass manufacturing. Electrochemical anodization of crystalline wafers in toxic acidic environment has been employed to produce porous silicon^27^. The silicon wafers have also been etched into tunable silicon nanowires via metal-assisted templated and non-templated approaches^28, 29^. However, the high-cost electronic grade wafers coupled with the milligram-per-wafer yield of active material limit the large-scale production on industry level. The hydrolysis of tetraethyl orthosilicate (TEOS) to produce nano-SiO_2_ with subsequent reduction into Si has been investigated to generate the high performance anode materials^30^. However, the extensive procedure to achieve TEOS as SiO_2_ precursor is inefficient for industry-level manufacturing.

Magnesiothermic reduction has been demonstrated as a morphology-protected method to reduce SiO_2_ into nanostructured silicon due to its relatively low operating temperature (~650 °C, <melting point of Si)^31, 32^. In comparison, carbothermal reduction is used to produce metallurgical silicon at a very high temperature (>2000 °C)^33^. This high energy-consuming process melts or liquefies Si, which destroys the original morphology of SiO_2_. Luo *et al*. have shown that the addition of NaCl effectively scavenges a large amount of heat generated during the highly exothermic reaction, which prevents the exceeded heat surpasses the melting point of Si^34^. Qian *et al*. have demonstrated that the incorporation of a molten salt of AlCl_3_ to SiCl_4_ decreases the reduction temperature to 200 °C, which preserves the original structure of SiO_2_^35^. Recently, silica derived from natural resources, such as rice husks^36^, beach sand^37^ and reed leaves^38^ have been reduced into porous Si via Mg-reduction as anode materials with excellent electrochemical performance. However, the strong acid leaching and high-temperature annealing process to remove metal ions and organic species contained in the natural precursors are time-consuming and energy-intensive, while large quantities of liquid and gaseous waste during etching and heating are produced. Also, the yield of SiO_2_ is low after the whole extraction and purification process. In this work, glass bottles are used as the SiO_2_ precursor to achieve high-purity Si with several advantages compared with the aforementioned silica sources above: (1) Glass bottles are directly utilized for reduction without pre-leaching and annealing, which offers a more environmentally-benign, energy-saving and efficient route to prepare silica. (2) Glass bottles are easily-collected and their abundance in silica without any loss due to the non-etching process result in the high yield of SiO_2_ as the reaction precursor. (3) Many tons of non-recycled glass ends up in the landfills aggravating the burden of waste disposal. This work provides a facile and green avenue to convert glass waste to beneficial materials. Accordingly, glass bottles are directly converted into high purity and interconnected Si network, and the carbon coated gSi exhibits stable cycling performance and high rate capability as anode material for LIBs in this work. We further design a Li-ion full cell using gSi@C anodes and LiCoO_2_ cathodes. The full cell demonstrates good initial cycling performance with high energy density. Compared with reported routes for the preparation of SiO_2_ (Fig. 1a), quartz powder derived from glass bottles can be directly used for the reduction process without pre-leaching in toxic acid and removing organic impurities at very high temperature. The content of quartz in glass is higher than those obtained from the majority of natural substances. In addition, many tons of un-recycled glass bottles produced every year may satisfy the demand for anode materials necessary in some energy storage applications. The Mg reduction process is conducted at a relatively low temperature, which inherits the original structure of the silica obtained from crushed glass bottles application. The overall process is facile, cheap and scalable for large scale fabrication of anode materials.

## Results

Silica as a common fundamental constituent obtained from sand is melt together with several minerals at high temperature to form the non-crystalline amorphous glass. Based on the glass ingredients including silica content and mineral components, glasses are primarily classified as fused silica glass, soda-lime-silica glass, sodium borosilicate glass and lead-oxide glass^39^. The usages of glasses are in food containers, housing and building, electronics and appliance, etc. Here we collect beverage glass bottles (corresponding to the soda-lime-silica type of glass with a high SiO_2_ content of 72%) as the quartz source in Fig. 1b. A glass bottle is put in thick bags and crushed into raw quartz (Fig. 1c, left). To reduce the size of quartz, mechanical milling in an alumina mortar is followed to downsize the raw SiO_2_ quartz to micrometer scale within minutes. The milled quartz powder is then transferred into tubes and dispersed in isopropanol (IPA) by ultra-sonication, which breaks the agglomeration while reducing the quartz into smaller size with nanometer and micrometer scale. The dispersion is then left referred to the settling process. The massive quartz particles precipitate to the bottom, while the lightweight quartz particles are small enough to remain suspended in IPA. These suspended particles are collected and assume a bright white appearance in stark (Fig. 1c, middle). Compared with the quartz sources derived from natural substances, the resultant glass powder is directly used as SiO_2_ precursor without leaching and annealing process. This simple and straight route to achieve relatively high-purity SiO_2_ is favorable for large-scale production.

The dried glass powder is mechanically milled and grounded with sodium chloride (NaCl, >99.5%, Fisher Scientific) in a weight ratio of 1:10 (w/w). The incorporation of NaCl acts as an effective heat scavenger to halt the reaction temperature rise at 801 °C during fusion, which assists in preserving the morphology of SiO_2_ particles below its melting point as illustrated in Fig. 1d. The well-mixed SiO_2_:NaCl powder is immersed in DI H_2_O and ultrasonicated for 2 hours with subsequent vigorously stirring at 60 °C for 3 hours. The solution is then dried at 105 °C in vacuum oven to remove the water. Dried SiO_2_:NaCl is grounded to pulverize NaCl crystals and mixed with Mg (99.5%, #325 mesh, Sigma Aldrich) powder in a 1:0.83 SiO_2_:Mg weight ratio followed by vortexing for ample mixing. The resultant powder is loaded into a SS 316 Swagelok-type reactor in Ar-filled VAC Omni-lab glovebox (H_2_O < 0.5 ppm, O_2_ < 0.5 ppm), and then immediately loaded into a MTI GSL-1200X 1″ diameter quartz tube and purged with argon. The reactor is heated to 700 °C at a heating rate of 5 °C and held for 6 hours to ensure the complete reduction of SiO_2_. After cooling down to room temperature, the resulting product is first washed with DI H_2_O several times to remove NaCl and then etched with 2 M HCl for 2 hours under stirring to remove excessive Mg, unwanted Mg_2_Si and MgO. The MgCl_2_ produced after etching can be recycled back to Mg by electrolysis, which is a sustainable process for the reproduction from waste to raw material^40^. The dispersion is centrifuged and further etched with 2 wt.% HF in tubes to remove the unreacted SiO_2_. The gSi powder is finally rinsed several times with DI H_2_O and ethanol, and dried under vacuum for 6 hours at 105 °C (Fig. 1c, right). The yield of gSi reduced from glass powder is close to the theoretical yield value of 46.7 wt.% (see supporting information), which offers an option for Si production on the industry level.

**Figure 2:**
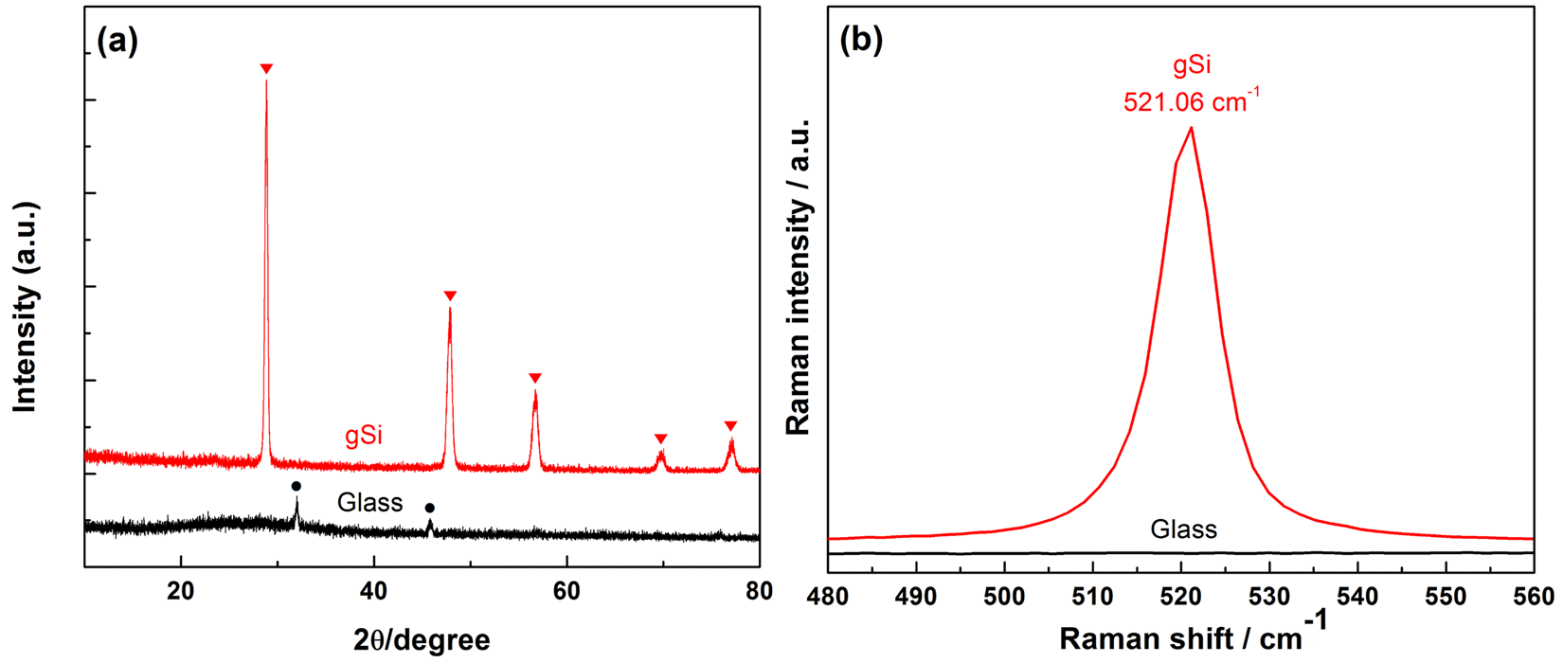
(**a**) XRD patterns and (**b**) Raman spectra of pre-reduction milled glass powder and post-reduction gSi.

The purities and phases of small size glass powder and as-reduced gSi are examined by X-ray diffraction (XRD) measurements in Fig. 2a. The weak XRD peaks associated with glass powder (index as black spectra) indicate the as-prepared quartz powder comprises minor by-products. The red XRD spectra of gSi demonstrates narrow and sharp peaks without amorphous scattering, suggesting the successful reduction from glass quartz to high degree crystallinity of Si. The peaks at 2θ of 28.8°, 47.8°, 56.7°, 69.7° and 77.1° represent (111), (220), (311), (400) and (331) planes, respectively^41^. Raman spectroscopy is carried to further verify the compositions of glass powder and gSi. The sharp peak at 521.06 cm^−1^ signifies the relatively high-purity of as-reduced gSi in Fig. 2b ^30^.

**Figure 3:**
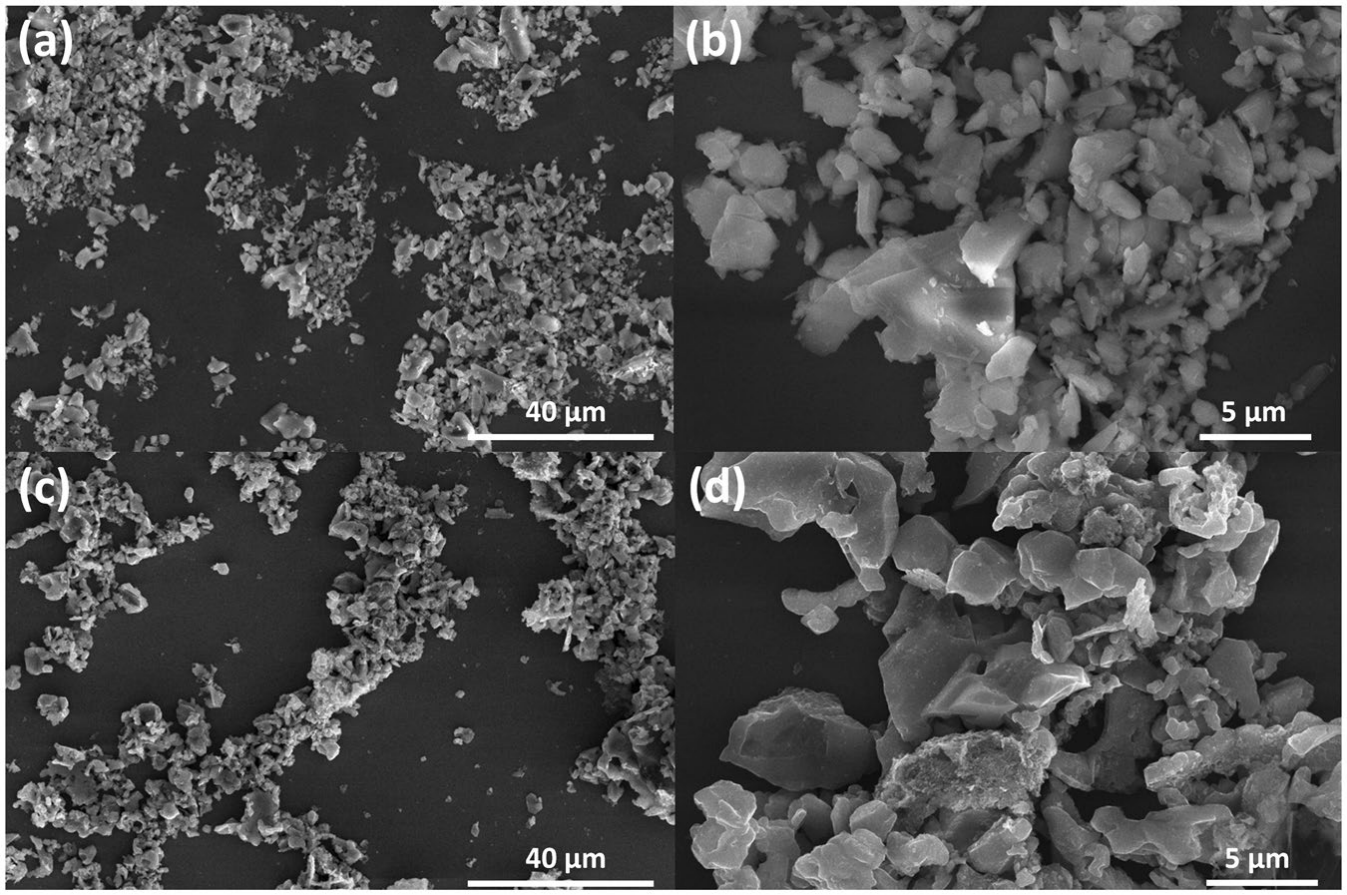
(**a**) Low magnification and (**b**) High magnification SEM images of milled glass powder. (**c**) Low magnification and (**d**) High magnification SEM images of gSi after reduction and acid-etching.

Scanning electron microscopy (SEM) micrographs are used to describe the structures and morphologies of the glass powder and gSi. The milled SiO_2_ powder displays a highly irregular-shape morphology with the particle size ranging from micrometer to nanometer scale as shown in Fig. 3a,b. The quantitative analysis obtained from the Energy Dispersive X-ray Spectroscopy (EDS) of quartz powder in Fig. S1 reveals the impurities may include lime (CaO), sodium oxide (Na_2_O) and alumina (Al_2_O_3_), which are the common mineral components for soda-lime-silica glass. After reduction, the particles remain irregular in shape owing to the morphology-protected Mg reduction process at a relatively low reaction temperature. The gSi has slightly reduced size distribution and partial porosity existing in gSi compared with the solid bulk SiO_2_ in Fig. 3c,d. This resulting cross-linked gSi networks with void spaces are attributed to the breakdown of the large particles during reaction, while acid-etching to remove MgO and Mg_2_Si within the original solid structure. EDS in Fig. S2 shows the weight occupancy of elements present in the gSi. The quantitative analysis reveals Si is the most dominant element a low residue of Mg, and the absences of metallic impurities imply that those oxides are reduced by magnesium, and then etched by HCl and HF. The existence of carbon in reduced silicon may increase the network conductivity for battery applications.

**Figure 4:**
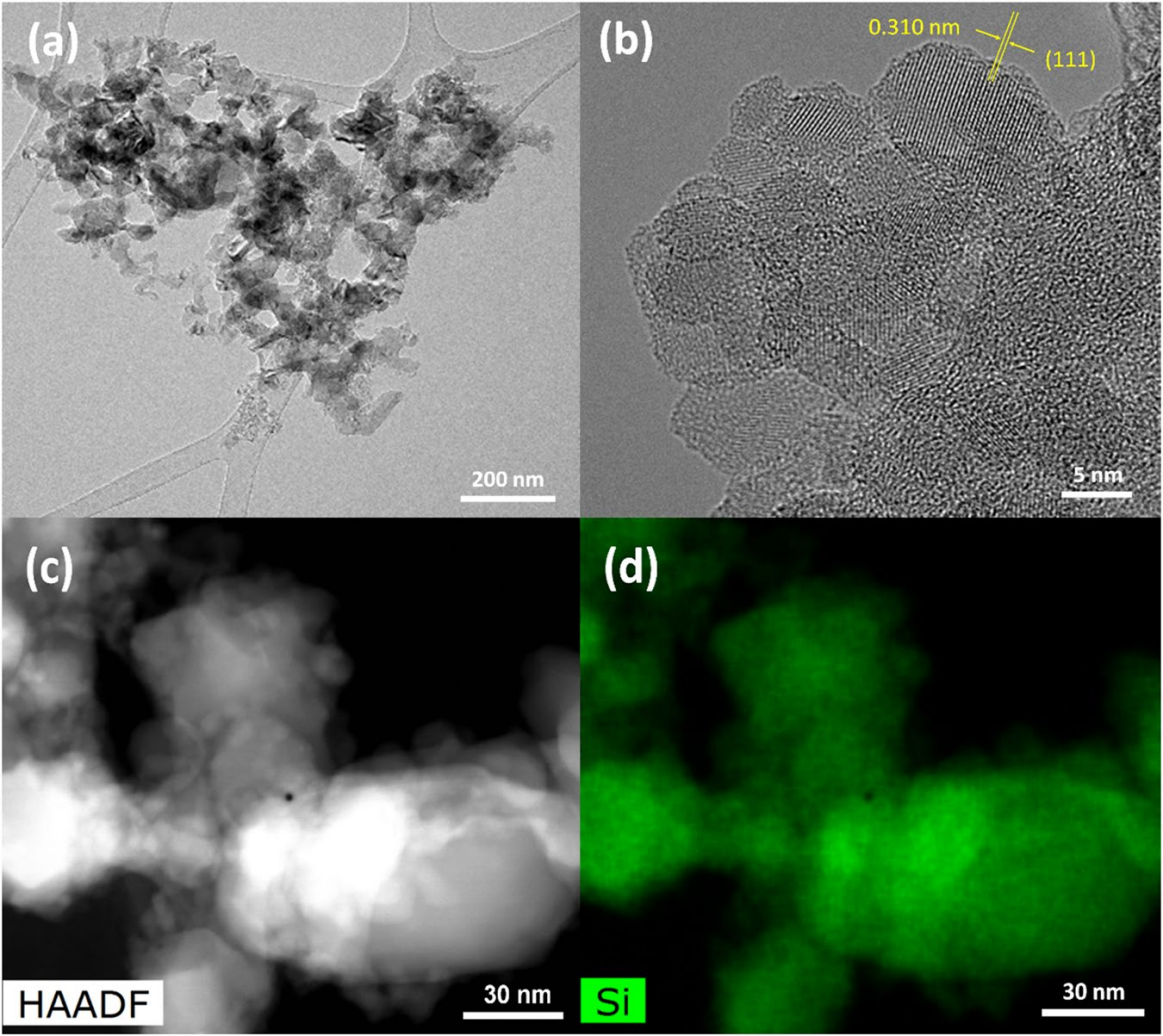
(**a**) Low magnification TEM image of gSi particles. (**b**) HRTEM image of gSi showing the characteristic lattice spacing of Si (111). (**c**) STEM-HAADF image of gSi and (**d**) EDS elemental map showing the high purity of reduced Si.

**Figure 5:**
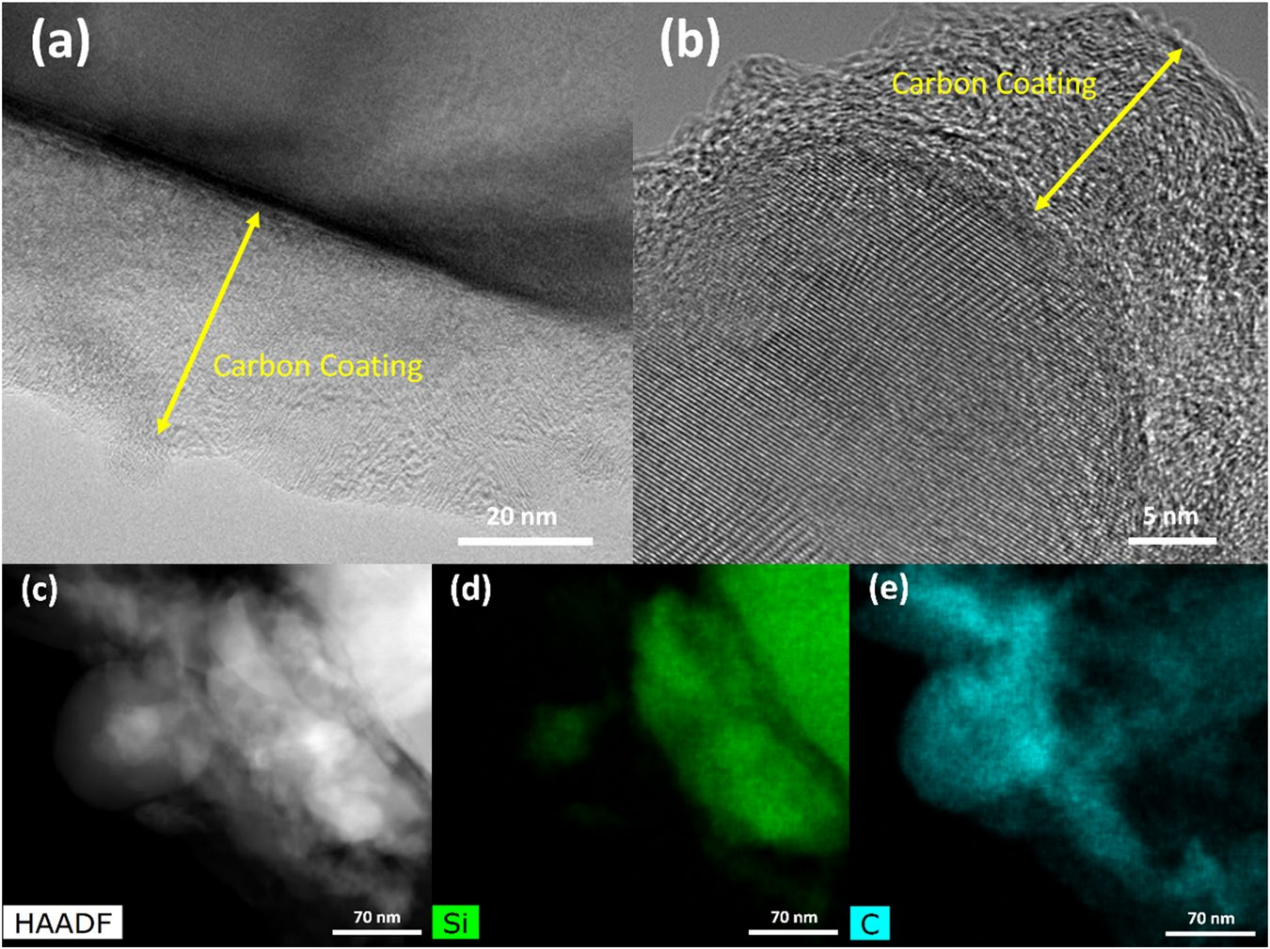
TEM images of gSi@C displaying the conformal carbon coating and the carbon layer thickness across gSi particles with (**a**) low magnification and (**b**) high magnification. (**c**) STEM-HAADF image of carbon coated gSi particles. (**d**,**e**) EDS mapping showing the phase conformal carbon coating surrounding gSi.

Transmission electron microscope (TEM) is carried out to further examine the structural information of glass silicon. Figure 4a confirms the existing large sparingly gSi comprises interconnected 3D gSi networks with the particle size from ~50 nm to micro level rather than the solid structure. This bridge-like interconnectivity is created by the selective removal of embedded MgO and Mg_2_Si in the gSi particles via acid etching. The existence of the partially internal porosity is available for buffering the volume expansion of Si during lithiation, while the SEI is well preserved, leading to less capacity decay resulting from the pulverization of active martials and the regeneration of SEI on exposed Si^42^. High-resolution TEM (HRTEM) and selected area electron diffraction pattern signify the highly crystalline nature of the gSi due to the d-spacing of 0.310 nm as shown in Fig. 4b and S3. Scanning transmission electron microscope (STEM) verifies the small particle size of the connected Si, while the high-angle annular dark-field imaging (HAADF) demonstrates the high purity of the reduce Si in Fig. 4c,d. Despite some void spaces generated by gSi network can accommodate volume change during lithiation, the low conductivity of Si limits its fast charge-discharge capability^43^. Thus, a conformally amorphous carbon coating with a thickness of 8–25 nm is introduced across all surfaces of gSi particles via CVD as shown in Fig. 5a,b. The gSi powder is loaded in a quartz boat and placed in the center of quartz tube furnace purged with Ar/H_2_ mixture. Acetylene (C_2_H_2_) is introduced to form the C-coating at 950 °C. The weight ratio of Si-to-C is ≈80:20 calculated by the weight variation before and after C-coating. STEM and HAAF of gSi@C clearly show the uniform distribution of carbon coating surrounding gSi, validating the successful deposition of the conformal carbon layer surrounding gSi in Fig. 5c–e. The seamless connection between C-coating and gSi improves the electrical conductivity of gSi@C composite while mitigating the volume change of silicon by the carbon buffer shell.

**Figure 6:**
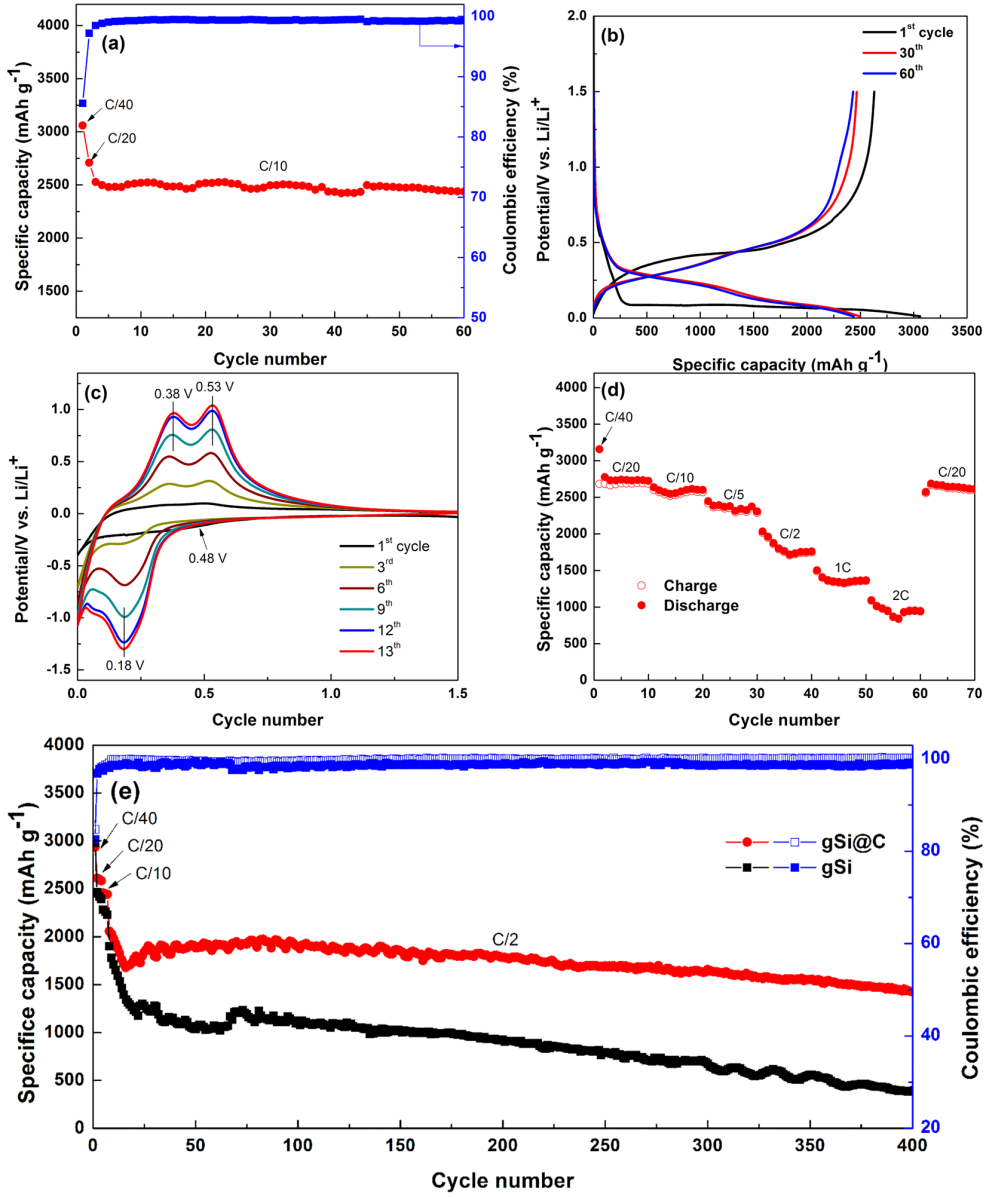
(**a**) Cycling performance and Coulombic efficiency of gSi@C anodes at a current density of C/10. (**b**) The corresponding galvanostatic charge-discharge profiles of gSi@C based half cell. (**c**) Cyclic voltammetry characteristic of gSi@C anodes. Scan rate: 0.2 mV sec^−1^. (**d**) C-rates cycling performance of gSi@C electrodes. (**e**) Comparison of the cycling performance and Coulombic efficiency between gSi@C and gSi based electrodes at a higher current density of C/2.

Button-type half-cell batteries were assembled in an Ar-filled glove box (O_2_ < 0.10 ppm, H_2_O < 0.5 ppm) with gSi@C as the anode material and pure Li-metal as the counter-electrode. Anode electrodes comprised 70 wt.% gSi@C as active material, 20 wt.% PAA as binder, and 10 wt.% carbon black as conductive additive. PAA has been proven to be an effective binder system for long-term cycling compared with PVDF and CMC due to its good mechanical properties and higher concentration of carboxyl groups to bind Si nanoparticles^44^. A porous polypropylene membrane (Celgard 3501) was used as the separator. The electrolyte contained 1 M LiPF_6_ dissolved in a mixture of fluoroethylene carbonate (FEC) and dimethyl carbonate (DMC) in a volume ratio of 1:1. The gSi@C electrodes demonstrated a discharge capacity of 2936 mAh g^−1^ with a Coulombic efficiency of 85% at C/40 (1 C = 3.6 A g^−1^) for the 1^st^ cycle in Fig. 6a. After the 2^nd^ cycle at C/20, the battery showed consistent current-potential behaviors for the subsequent cycles at C/10. The capacity faded very slightly and demonstrated a capacity ~2500 mAh g^−1^ over 60 cycles as supported in Fig. 6b. The Coulombic efficiency calculated from all the cycles excluding the 1^st^ cycle is >99%, which suggests the excellent reliability and reversibility for the gSi@C half cells.

Cyclic voltammetry (CV) was tested in a voltage window range of 0.01 to 1.5 V (vs. Li^+^/Li) with a scan rate of 0.2 mV sec^−1^ as shown in Fig. 6c. The first discharge curve showed a weak peak around 0.48 V and disappeared in the subsequent cycles, which demonstrated the Li-ions were consumed to form stable SEI components (Li_x_SiO_y_, lithium ethylene discarbonate and LiF) and leads to the irreversible charge capacity in the first cycle^45^. The peaks (0.18 V and 0.10 V) associated with lithiation and the peaks (0.38 V and 0.53 V) corresponding to delithiation increased and coincided at the 12^th^ and 13^th^ cycles, which suggested a kinetic enhancement process for stabilizing active materials^37^. Conformal C-coating has been verified to be an effective route to improve cycling stability and rate capability^15^. Rate capability of gSi@C and gSi anodes were demonstrated with different current densities measured from C/20 to 2 C in Fig. 6d. Even up to 2 C, the gSi@C electrodes showed a much higher capacity of ~1000 mAh g^−1^ over that of gSi electrodes, which signified the substantial enhancement of conductive coatings on rate capability of Si anodes. The charge-discharge profiles of gSi@C and gSi electrodes under various C-rates are shown in Fig. S4a and c, respectively. For long-term cycling, the gSi@C electrodes were properly activated at 40/C, 20/C and 10/C at the initial cycles to achieve a stable SEI as confirmed by CV measurements (Fig. 6e). After the kinetic enhancement was completed at low current densities, the gSi@C anodes were cycled at a higher C-rate of C/2 and exhibited a reversible capacity of ~1420 mAh g^−1^ with capacity retention of 72% (Coulombic efficiency of >99.5%) after 400 cycles. In comparison, non-carbon coated gSi electrodes presented a lower capacity of 796 mAh g^−1^ with capacity retention of 20% (corresponding to Coulombic efficiency of 98.9%) after the same cycling process. The cycling results of gSi@C electrodes are comparable to several reported Si anodes via Mg-reduction. For instance, silicon electrodes obtained from diatomaceous earth showed fast decay from 1400 to 700 mAhg^−1^ after 30 cycles^46^. Reed leaves and rice husk derived Si based anodes demonstrated capacities of ~1050 and ~1580 mAhg^−1^ with capacity retentions of 50% and 86% at C/2 after 200 cycles and 300 cycles, respectively^36, 38^. The gSi@C half-cells exhibited ~1790 and ~1660 mAhg^−1^ (corresponding to capacity retentions of 91% and 84%) after 200 and 300 cycles at C/2, respectively, which are similar or even slightly superior over the mentioned silicon anode performances. The slopes of discharge-discharge curves of amorphous Li-Si are in good agreement with the peaks of CV curves in Fig. S4b and d ^12^. This performance difference between gSi@C and gSi anodes was mainly due to the low conductivity of non-carbon coated gSi, leading to insufficient charge transfer between gSi and the micro-level carbon black within the electrode. Moreover, the carbon coating surrounding gSi improved the cycling stability of anodes, which was confirmed by the higher capacity retention and Coulombic efficiency of gSi@C electrodes over that of pure gSi after 400 cycles.

**Figure 7:**
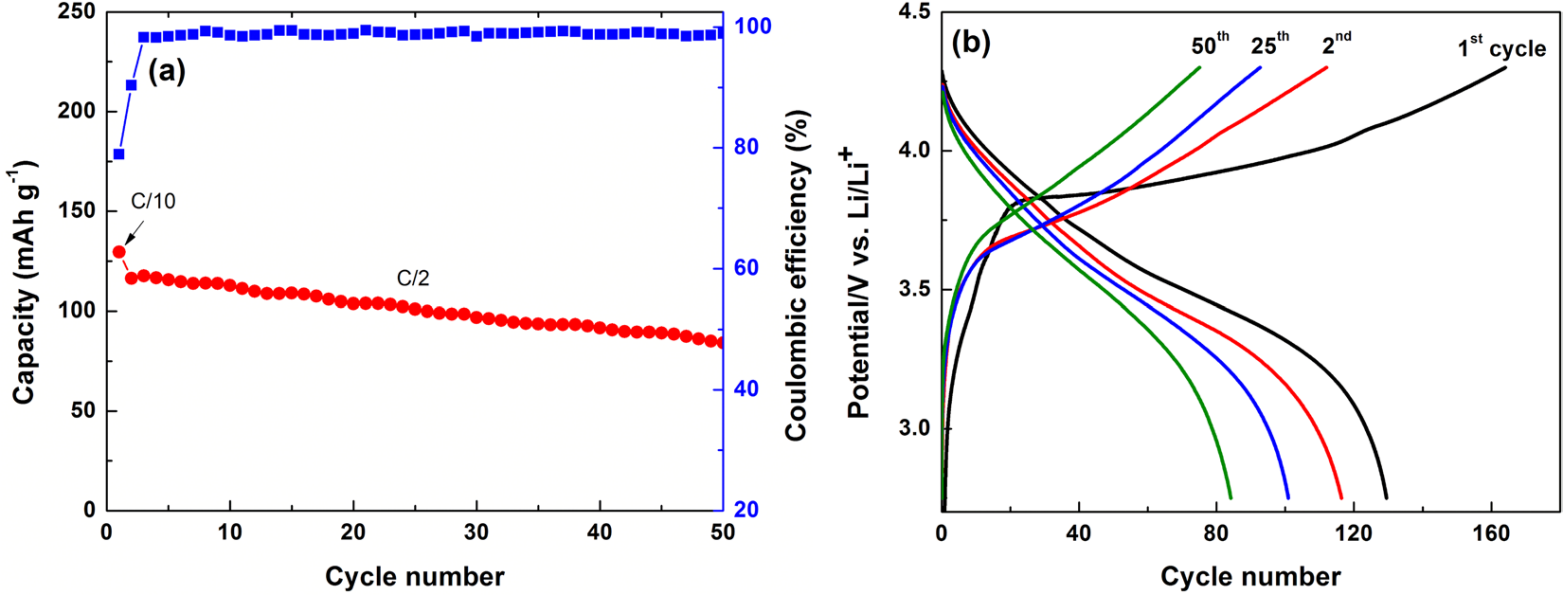
(**a**) Cycling performance of full cells employing gSi@C anode and LiCoO_2_ cathode for 50 cycles. (**b**) The corresponding galvanostatic charge-discharge curves of the full cells.

To further characterize the practical capability of the as-prepared gSi anodes, a full cell comprising gSi@C as anode and LiCoO_2_ (provided by TET USA Corporation) cathode was fabricated. In an aim to achieve a theoretically optimal total capacity of a full cell, the capacity ratio of anode to cathode is expected to be close to 1:1^47, 48^. In this work, the gSi@C anodes with a Si loading of ~0.5 mg cm^−2^ demonstrate a reversible capacity of ~1800 mAh g^−1^ at C/2 (1 C = 3.6 A g^−1^) over the first 50 cycles, while the LiCoO_2_ cathodes with a mass loading of ~5.4 mg cm^−2^ show a reversible capacity of ~160 mAh g^−1^ at C/2 (1 C = 163 mA g^−1^). The practical capacities of gSi@C and LiCoO_2_ in half cells are calculated to be 0.900 mAh cm^−2^ mAh and 0.865 mAh cm^−2^. Accordingly, the actual capacity ratio of Si anode to LiCoO_2_ cathode is 1.04:1, which suggests this gSi@C/LiCoO2 full cell design is suitable for evaluation of anode and cathode effects on cycling stability. The slightly higher Si content is favorable to prevent the anodes from over-lithiated during full-cell charge. The cycling performance of the full cell is measured with a voltage window range from 2.7 to 4.3 V as shown in Fig. 7a. The initial cycle is tested at C/20 for the proper activation of active materials, and a total capacity of ~130 mAh g^−1^ (corresponding to a Coulombic efficiency of 78.9% in Fig. S4b) is displayed. Note that the capacity of full cell is calculated only based on the total mass of Si and LiCoO_2_. In the subsequent cycles, the capacity gradually decreases from 116 to 85 mAh g^−1^ at C/2 until the 50^th^ cycle. An energy density of 460 Wh kg^−1^ is demonstrated by this full cell for the first cycle. Even after 50 cycles, the cell assembly still exhibits an energy density of 298 Wh kg^−1^, which is comparable to those of reported full cells based on Si anodes^47, 49, 50^. If the masses of binders (PAA, polyvinylidene fluoride), carbon black and current collectors (Cu and Al foils) for gSi@C anodes and LiCoO_2_ cathodes are also included, the full cell shows an energy density of 139 Wh kg^−1^ for the 1^st^ cycle with 65 Wh kg^−1^ over 50 cycles (Table S1).

## Discussion

Electrochemical impedance spectroscopy (EIS) was used to characterize the electrochemical performance of the gSi@C anodes. A small sinusoidal of 10 mV was applied to gSi@C anodes and the resultant complex impedance was measured in a frequency range between 100 mHz and 1 MHz. The impedance information was modeled using an electrical equivalent circuit shown in Fig. S5a. The real axis interception at high frequency, also known as equivalent series resistance (R_s_ or ESR), denotes the ionic resistance of the electrolyte in summation with the electronic resistance of the active material within the electrode. R_s_ declines for the first 7 cycles and then stabilizes thereafter in Fig. S5b and Table S2. The high frequency semicircle represents the resistance of SEI layer (R_SEI_) coupled with resistance stemming from the imperfect contact between metal current collector and active materials (R_INT_)^51, 52^. The R_SEI+INT_ decreases in diameter as the cycle increases, while the semicircle at mid frequency drops sharply for the first 7 cycles and then stabilizes, indicating the stabilization of charge transfer impedance as shown in Fig. S5c. Interfacial impedance remains constant as cycling, which signifies the contact impedance among active particles and current collect is independent with cycling^53^. This phenomenon can be attributed to the buffering effect of carbon coating on the Si volume expansion^54^. Besides the high frequency semicircle (corresponding to R_SEI+INT_) and the mid semicircle (owing to charge transfer impedance between electrolyte and electrode), another distinct arc, known as the low-frequency (<20 MHz) Warburg impedance tail, represents impedance resulting from diffusion of ions into the active materials^55, 56^. This parameter is related to the diffusion of lithium into gSi@C and salt transfer in the electrolyte. The arcs show big difference for the first 7 cycle, while the tails of 8^th^ and 9^th^ closely overlap, confirming the anodes tend to stabilize as cycling.

Effective strategies have been utilized to alleviate the volume change of gSi particles during lithiation/delithiation and improve the cycling stability. First, the addition of a large amount of NaCl to glass quartz power produces cross-liked structure during the highly exothermic magnesium reduction process. The reaction process generates a large amount of heat as shown in Equations 1–2 as follows^33^:

1 $$2{\rm{Mg}}({\rm{g}})+{{\rm{SiO}}}_{2}({\rm{s}})\to {\rm{Si}}({\rm{s}})+2{\rm{MgO}}({\rm{s}}),\,{\rm{\Delta }}{\rm{H}}=-546.42\,{\rm{kJ}}\,\,{{\rm{mol}}}^{-1}.$$

Mg is in excess in this work,

2 $$2{\rm{Mg}}({\rm{g}})+{\rm{Si}}({\rm{s}})\to {{\rm{Mg}}}_{2}{\rm{Si}}({\rm{s}}),\,{\rm{\Delta }}{\rm{H}}=-318.91\,{\rm{kJ}}\,{{\rm{mol}}}^{-1}.$$

The continuously feeding heat can cause the fusion of silicon, which destroys its original morphology and leads to aggregation of Si particles. However, the NaCl is used to cover SiO_2_ particles and behave as heat scavenger to absorb a large amount of heat for self-fusion, which has been verified to effectively protect the surface morphology of Si after reduction. NaCl is low cost, non-toxic and easily recycled to use. The etching of byproduct MgO and Mg_2_Si within Si structure generates the interconnected Si network with empty spaces, which mitigates volume change of silicon during alloying and improves Li-ions transfer into the active material. Second, a conformal carbon coating on the surface of gSi not only acts as buffer layer for accommodating volume expansion, but also enhances the rate capability of the electrodes. Moreover, the FEC-containing electrolyte assists the formation of SEI thin films with superior surface properties on Si particles compared to the thicker films formed in FEC-free electrolyte, and the presence of FEC lowers the impedance in electrolyte solutions, which reduces the irreversible capacity of the electrodes^57^.

## Conclusion

In summary, we have demonstrated the direct conversion from glass bottles to high purity silicon using a scalable, facile and low-cost Mg reduction process. The excellent electrochemical performance of gSi@C anodes can be mainly attributed to the mitigated volume expansion and improved system conductivity resulting from the interconnected gSi network and the conformal carbon coating. A full cell with good initial cycling stability and high energy density using gSi@C as anode and LiCoO_2_ as cathode has been reported. The non-etching, easy-collet and abundant glass bottles as SiO_2_ source offers a promising avenue for the large-scale production of Si based anodes.

## Methods

### Materials synthesis

Collected beverage glass bottles were first sealed in several thick bags and crushed into small pieces by hammer. Crushed glass was hand-milled in an alumina mortar for several minutes, transferred into tubes with ultrasonication for 2 hours in isopropanol (IPA), and then left for settling big quartz down for 2 hours. Light-weight suspended quartz particles in IPA were collected and dried at 90 °C under vacuum for 2 hours. To sufficiently utilize the raw materials, the left big quartz were further milled into small size particles later. Dried small glass powder is mixed with NaCl (>99.5%, Fisher Scientific) in a weight ratio of 1:10 (3 g:30 g, w/w) and milled in an alumina mortar. The well-mixed SiO_2_:NaCl powder was added in DI H_2_O and ultrasonicated for 2 hours with subsequent stirring for 3 hours. The mixture was dried overnight at 105 °C in vacuum oven to remove water. The resulting SiO_2_:NaCl powder is grounded with Mg (99.5%, #325 mesh, Sigma Aldrich) in a weight ratio of 1:0.83 (Si:Mg: 3 g:2.49 g, w/w) ratio. The well-mixed powder was loaded into SS 316 Swagelok-type reactors in argon-filled VAC Omni-lab glove box (<0.5 ppm H_2_O, < 0.5 ppm O_2_). The reactors were loaded into MTI GSL-1200X quartz tube furnace and purged with argon. The furnace was ramped to 700 °C with a heating rate of 5 °C min^−1^, held for 6 hours at 0.5 sccm argon environment and cooled to room temperature. The resultant powder was washed with DI H_2_O and ethanol several times to remove NaCl, followed by etching unwanted MgO and Mg_2_Si in concentrated HCl with subsequent washing with DI H_2_O. Unreacted SiO_2_ is removed by etching in 5% HF for 1 hour and washed with DI H_2_O and ethanol, and dried under vacuum for 4 hours at 90 °C. The yield of high-purity gSi derived from glass powder is 40.0–40.3 wt.%. Thin carbon layer coated gSi is formed by CVD. The dried and milled gSi powder is loaded in a quartz boat and transferred into the center of a quartz tube furnace. 30 sccm C_2_H_2_ is introduced and carried by Ar/H_2_ (150/50, sccm) to produce the amorphous carbon coating across gSi surface at 950 °C for 15 minutes. Si-to-C weight ratio is calculated to be 4:1 based on the weight variation before and after carbon coating.

### Materials characterization

The surface morphology is investigated using optical microscopy, scanning microscopy (SEM; Leo-Supra, 1550) with an X-ray energy-dispersive spectroscopy (EDS). Transmission electron microscopy (TEM, Titan Themis 300) operated at 300 KV is used to further characterize the purity and morphology of gSi and gSi@C. The TEM samples are prepared by dispersing the powder in water for 15 minutes, diluted and then dropped onto TEM grids. The phase identification is performed by X-ray diffraction (XRD, PANalytical Empyrean) from 10° to 80°. Raman spectroscopy (Renishaw DXR) with a 532 nm laser (8 mW excitation power, 100X objective lens) source is carried to check the purity of gSi. Electrochemical impedance spectroscopy (EIS) analysis is obtained with a Biologic VMPs.

### Electrochemical measurements

The anode electrodes were prepared by doctor-blading a slurry on pre-cleaned Cu foil with a pre-area mass loading for 0.5–0.6 mg cm^−2^. The slurry comprises 70% active material (gSi@C), 20% PAA binder and 10% conductive additive (carbon black). A button-type (CR 2032) half-cell configuration was used for the electrochemical measurements. Cells were assembled in an Argon-filled VAC Omni-lab glovebox with oxygen and H_2_O level below 0.5 ppm. Pure Li metal was used as the counter electrode for half-cell tests. Full cells were prepared and evaluated in TET USA Corporation facility with custom made LiCoO_2_ (lithium cobalt oxide) cathodes with a LiCoO_2_ mass loading of 5.3–5.5 mg cm^−2^. A Celgard 3501 porous PP membrane was used as the separator. The electrolyte comprising 1 M LiPF_6_ in fluoroethylene carbonate and dimethyl carbonate (FEC:DMC = 1:1, v/v) was used as electrolyte for half and full cells. Cycling performance and galvanostatic charge-discharge behaviors were conducted on Arbin BT300 with a voltage window ranging from 0.01 to 1.5 V (vs. Li^+^/Li). Capacity and C-rates were determined using 1 C = 3.6 A g^−1^. Cyclic voltammetry scans were tested at a fixed voltage window between 0.01 V and 1.5 V (vs. Li^+^/Li). Electrochemical impedance spectroscopy measurements were performed to evaluate the impedance information of gSi@C anodes on a Biologic VMPs with a frequency range between 0.01 Hz and 1 MHz.

## Electronic supplementary material

Supplementary Information
