# Supplementary material for: Silicon Derived from Glass Bottles as Anode Materials for Lithium Ion Full Cell Batteries
Source: Sci Rep. 2017 Apr 19;7:917. doi: 10.1038/s41598-017-01086-8 (PMC5430423; doi:10.1038/s41598-017-01086-8)
Supplement: Supplementary file 1 — Supplementary Information [file 41598_2017_1086_MOESM1_ESM.pdf]

# Supporting Information

## Silicon Derived from Glass Bottles as Anode Materials for Lithium Ion Full Cell Batteries

**Changling Li[1], Chueh Liu[2], Wei Wang[1], Zafer Mutlu[1], Jeffery Bell[1], Kazi Ahmed[1], Rachel Ye[2], Mihrimah Ozkan[2]*, and Cengiz S. Ozkan[1]***

**Yield of gSi reduced from glass powder:**

Based on equation 1: $2Mg(g) + SiO_2(s) \rightarrow Si(s) + 2MgO(s)$:

Theoretical Si yield = (28.085 g/mol)/ [(28.085 + 15.992 x 2) g/mol] = 0.467 = 46.7 wt%

In this work, 1.2-1.3 g high-purity Si is derived from 3 g glass powder (corresponding to a yield of 40.0-40.3 wt.%), which is close to the theoretical yield value. The loss of Si can be attributed to the unreacted $SiO_2$, etching and washing process.

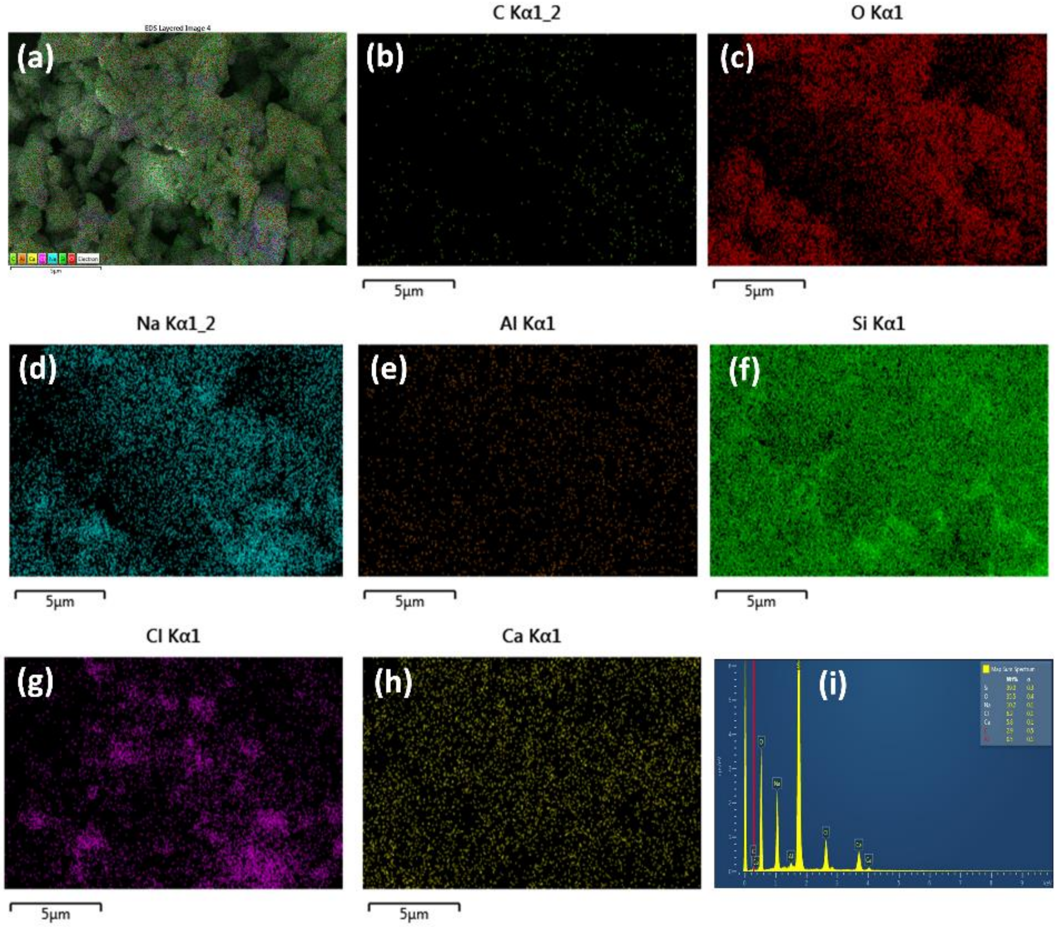


**Figure S1.** EDS elemental mapping of milled glass powder: a) Electron image with superposed elemental maps. b-h) Elemental maps of C, O, Na, Al, Si, Cl and Ca. i) Elements weight percentages (Si: 39.0%, O: 35.5%, Na: 10.2%, Cl: 6.2%, Ca: 5.8%, C: 2.9%, Al: 0.5% in weight).

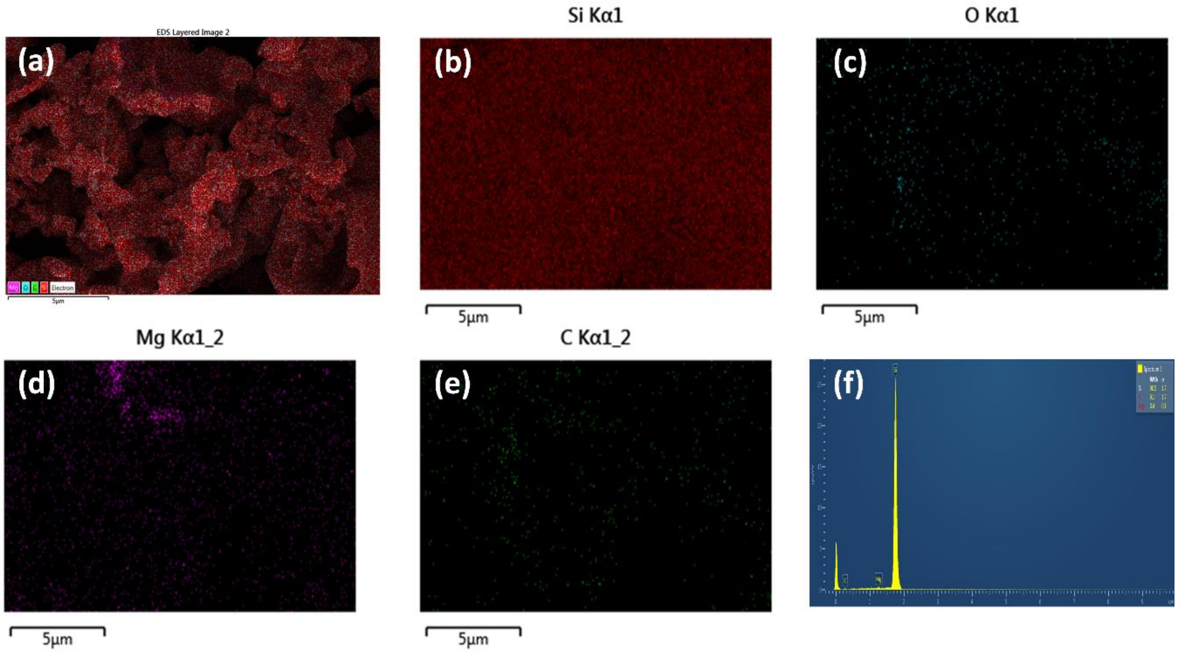


**Figure S2.** EDS elemental mapping of g-Si: a) Electron image with superposed elemental maps. b-e) Elemental maps of Si, O, Mg and C. f) Elements weight occupancies (Si: 90.5%, C: 9.1%, Mg: 0.4% in weight).

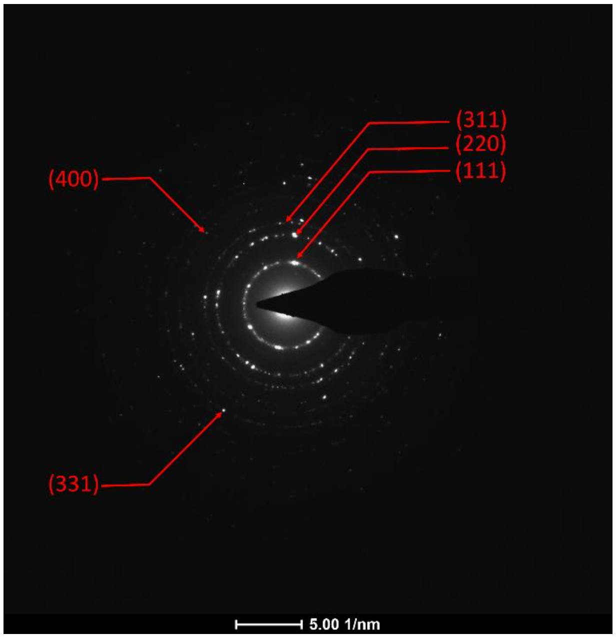


**Figure S3.** Selected area electron diffraction pattern of g-Si, showing the high purity of reduced Si material.

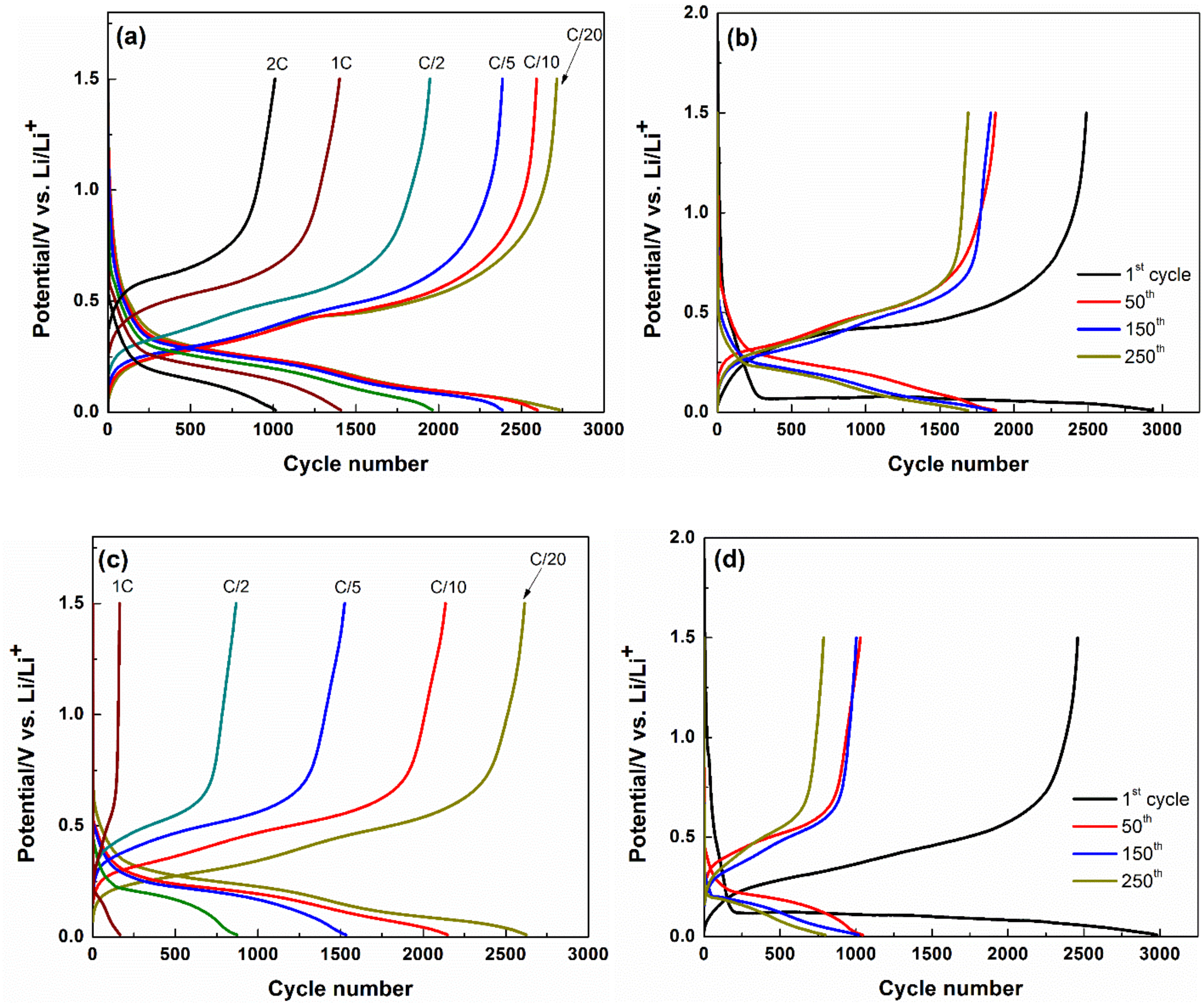


**Figure S4.** The corresponding charge-discharge profiles of C-rates measurements and cycling performance for (a-b) gSi@C electrodes and (c-d) g-Si electrodes.

**Table S1.** Mass portion of individual components used in pouch full cell.

| Component | Anode (gSi, PAA, CB) | Cu Foil | Cathode ($LiCoO_2$, PVDF, CB) | Al Foil | Total |
|---|---|---|---|---|---|
| Weight (mg) | 1.44 | 18.03 | 11.32 | 7.79 | 38.58 |
| Mass portion (%) | 3.73 | 46.73 | 29.35 | 20.19 | 100 |

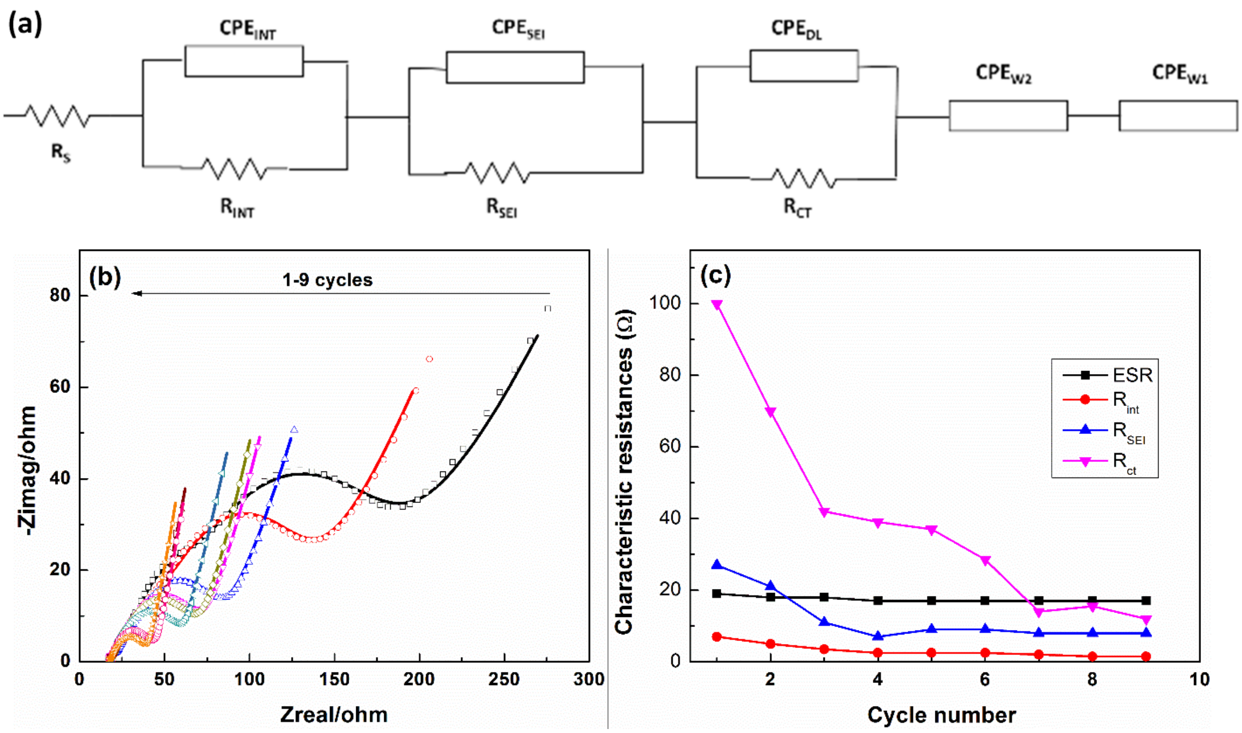


**Figure S5.** a) Equivalent circuit of gSi@C based anodes used to produce fitted model data. b) Nyquist plots of gSi@C half cell. c) Characteristic resistances for EIS measurements.

**Table S2.** Fitted equivalent circuit elements of gSi@C for 9 cycles.

| Cycles | ESR | $R_{int}$ | $R_{SEI}$ | $R_{ct}$ | $Q_{int}$ | $n_{int}$ | $Q_{SEI}$ | $n_{SEI}$ | $Q_{dl}$ | $n_{dl}$ | $Q_{w1}$ | $n_{w1}$ | $Q_{w2}$ | $n_{w2}$ |
|---|---|---|---|---|---|---|---|---|---|---|---|---|---|---|
| 1 | 19 | 7 | 27 | 100 | 0.000007 | 0.8 | 0.00002 | 0.83 | 0.00025 | 0.68 | 0.05 | 0.65 | 0.01 | 0.27 |
| 2 | 18 | 5 | 21 | 70 | 0.000007 | 0.77 | 0.00008 | 0.75 | 0.00035 | 0.75 | 0.05 | 0.65 | 0.015 | 0.3 |
| 3 | 18 | 3.5 | 11 | 42 | 0.000007 | 0.77 | 0.0001 | 0.75 | 0.00035 | 0.75 | 0.055 | 0.75 | 0.025 | 0.35 |
| 4 | 17 | 2.5 | 7 | 39 | 0.000007 | 0.77 | 0.00015 | 0.75 | 0.0004 | 0.71 | 0.055 | 0.8 | 0.03 | 0.4 |
| 5 | 17 | 2.5 | 9 | 37 | 0.000007 | 0.77 | 0.00015 | 0.75 | 0.00045 | 0.71 | 0.055 | 0.8 | 0.035 | 0.45 |
| 6 | 17 | 2.5 | 9 | 28.5 | 0.000007 | 0.77 | 0.0002 | 0.75 | 0.00055 | 0.71 | 0.06 | 0.8 | 0.04 | 0.5 |
| 7 | 17 | 2 | 8 | 14 | 0.000007 | 0.77 | 0.0003 | 0.7 | 0.0008 | 0.71 | 0.08 | 0.85 | 0.05 | 0.55 |
| 8 | 17 | 1.5 | 8 | 15.5 | 0.000007 | 0.77 | 0.0004 | 0.7 | 0.0008 | 0.7 | 0.09 | 0.9 | 0.055 | 0.55 |
| 9 | 17 | 1.5 | 8 | 12 | 0.000007 | 0.77 | 0.0004 | 0.65 | 0.001 | 0.7 | 0.09 | 0.9 | 0.055 | 0.55 |
